# Pulmozyme Ameliorates LPS-Induced Lung Fibrosis but Provokes Residual Inflammation by Modulating Cell-Free DNA Composition and Controlling Neutrophil Phenotype

**DOI:** 10.3390/biom15020298

**Published:** 2025-02-17

**Authors:** Ludmila A. Alekseeva, Aleksandra V. Sen’kova, Khetam Sounbuli, Innokenty A. Savin, Marina A. Zenkova, Nadezhda L. Mironova

**Affiliations:** 1 Institute of Chemical Biology and Fundamental Medicine, Siberian Branch of the Russian Academy of Sciences (SB RAS), Lavrentiev Ave., 8, Novosibirsk 630090, Russia; alekseeva.mila.23@yandex.com (L.A.A.); senkova_av@niboch.nsc.ru (A.V.S.); khetam.sounbuli.edu@gmail.com (K.S.); savin_ia@niboch.nsc.ru (I.A.S.); marzen@niboch.nsc.ru (M.A.Z.); 2 Faculty of Natural Sciences, Novosibirsk State University, Pirogova St., 1, Novosibirsk 630090, Russia

**Keywords:** lung inflammation, lung fibrosis, DNase I, cell-free DNA, neutrophils

## Abstract

Pulmonary fibrosis, a chronic progressive lung disorder, can be the result of previous acute inflammation-associated lung injury and involves a wide variety of inflammatory cells, causing the deposition of extracellular matrix (ECM) components in the lungs. Such lung injury is often associated with excessive neutrophil function and the formation of DNA networks in the lungs, which are also some of the most important factors for fibrosis development. Acute lung injury with subsequent fibrosis was initiated in C57Bl/6 mice by a single intranasal (i.n.) administration of LPS. Starting from day 14, human recombinant DNase I in the form of Pulmozyme for topical administration was instilled i.n. twice a week at a dose of 50 U/mouse. Cell-free DNA (cfDNA), DNase activity, and cell content were analyzed in blood serum and bronchoalveolar lavage fluid (BALF). Inflammatory and fibrotic changes in lung tissue were evaluated by histological analysis. The gene expression profile in spleen-derived neutrophils was analyzed by RT-qPCR. We demonstrated that Pulmozyme significantly reduced connective tissue expansion in the lungs. However, despite the reliable antifibrotic effect, complete resolution of inflammation in the respiratory system of mice treated with Pulmozyme was not achieved, possibly due to enhanced granulocyte recruitment and changes in the nuclear/mitochondrial cfDNA balance in the BALF. Moreover, Pulmozyme introduction caused the enrichment of the spleen-derived neutrophil population by those with an unusual phenotype, combining pro-inflammatory and anti-inflammatory features, which can also maintain lung inflammation. Pulmozyme can be considered a promising drug for lung fibrosis management; however, the therapy may be accompanied by residual inflammation.

## 1. Introduction

Pulmonary fibrosis is a chronic progressive lung disease characterized by increased deposition of extracellular matrix (ECM) components in the lung parenchyma, leading to disruption of lung tissue architecture and decreased functional activity [1]. Although idiopathic pulmonary fibrosis accounts for a significant proportion of pulmonary fibrosis cases, in some patients, the disease develops as a result of poorly controlled inflammation in the lungs, triggering regeneration mechanisms that can lead to increased synthesis of ECM components. At the cellular level, the key players in the development of pulmonary fibrosis are macrophages, which are the first immune cells to arrive at the site of tissue damage, and fibroblasts, cells of mesenchymal origin that, as fibrosis progresses, transform into myofibroblasts, which have motor activity and synthesize ECM components [2]. A distinctive feature of the development of pulmonary fibrosis is that the increasing “rigidity” of the pulmonary framework stimulates the extreme proliferation of myofibroblasts, which leads to an intensification of the synthesis of ECM components, thereby forming a “vicious circle” [3].

Another factor linked to the development of pulmonary fibrosis is neutrophils. Accumulated data confirm the key role of neutrophils in the development and progression of pulmonary fibrosis; NE (neutrophil elastase) and MMP (matrix metalloproteases) produced by neutrophils are involved in ECM remodeling [4,5]. Furthermore, NE promotes fibroblast proliferation, myofibroblast differentiation, and TGFb1 activation and stimulates the synthesis and deposition of ECM components [6].

Activated neutrophils release into extracellular space web-like structures consisting of sparse nuclear chromatin and cytosolic proteins, which are called neutrophil extracellular traps (NETs) [7,8,9]. A close relationship between NET formation and fibrosis development has been described for a number of organs, including the lungs. In particular, NETs have been detected in the sputum and lungs of patients with acute respiratory distress syndrome (ARDS) [10], chronic obstructive pulmonary disease (COPD) [11], neutrophil-associated asthma [11], and non-specific interstitial pneumonia [12]. The presence of NETs in the lungs of patients with non-specific interstitial pneumonia [10] with a subsequent increase in NET components and a decrease in the level of NET-degrading DNase I has also been reported in patients with polymyositis/dermatomyositis and interstitial lung disease [12,13]. Thus, NETs can be considered integral to the fibrotic process and a target for NET-derived DNA-degrading enzymes to reduce fibrosis.

The commercially available drug Pulmozyme is a human recombinant DNase I in a form adapted for inhalation that is used for sputum liquefaction to treat cystic fibrosis [14]. Repeated attempts have been made to use Pulmozyme for the treatment of other diseases, including cancer, in experimental laboratory animal models and preclinical settings [15,16,17].

In this study, we investigated the use of Pulmozyme for the treatment of LPS-induced pulmonary fibrosis in C57Bl/6 mice. Pulmozyme administered intranasally (i.n.) at a dose of 50 U/mouse reduced fibrotic changes in the lungs; however, signs of secondary inflammation were detected. It was shown that Pulmozyme elevated mitochondrial DNA sequences in cfDNA isolated from bronchoalveolar lavage fluid (BALF), which can maintain inflammation, and led to the enrichment of the spleen-derived neutrophil population by those with an unusual phenotype, combining pro-inflammatory and anti-inflammatory features.

## 2. Materials and Methods

### 2.1. Mice

We used male C57Bl/6 mice with an average weight of 25–30 g obtained from the vivarium of the Institute of Chemical Biology and Fundamental Medicine SB RAS (Novosibirsk, Russia). Mice were kept in plastic cages (nine animals per cage) under standard daylight conditions. Water and food were provided ad libitum. All animal procedures were performed in accordance with the recommendations for the proper use and care of laboratory animals (European Communities Council Directive 2010/63/EU). The experimental protocols were approved by the Ethics Committee for Animal Experiments of the Institute of Cytology and Genetics SB RAS (Novosibirsk, Russia) (Protocol No. 56, 10 August 2019 and No. 187, 3 October 2024).

### 2.2. Fibrosis Induction and Design of Animal Experiment

Lung inflammation with subsequent fibrosis was induced by the intranasal (i.n.) administration of LPS (O55:B5, Sigma-Aldrich, St. Louis, MO, USA) at a dose of 5 mg/kg in 50 µL of saline solution under isoflurane anesthesia [Laboratorios Karizoo, Barselona, Spain]. On day 14 after induction, Pulmozyme (dornaze alpha, 1000 U/mg, 1 mg/mL, Genotech Inc., South San Francisco, CA, USA) was administered i.n. at a dose of 50 U/mouse in 50 µL of saline solution. Mice were divided into the following groups: (1)—healthy mice; (2)—mice with LPS-induced lung inflammation/fibrosis receiving saline solution (control); and (3)—mice with LPS-induced lung inflammation/fibrosis receiving Pulmozyme.

On days 14, 21, 28, and 35 after induction, peripheral blood was collected from the retro-orbital sinus of mice during their lifetime, after which the mice were sacrificed, and the BALF, lung tissue, and spleen were collected for subsequent analysis.

### 2.3. Collection and Analysis of the Bronchoalveolar Lavage Fluid (BALF)

The lungs of mice were washed with 1 mL of a cold saline solution. The collected BALF samples were centrifuged at 1500 rpm and 4 °C for 10 min. The supernatants were collected for subsequent evaluation of cfDNA concentrations and DNase activity. The cell pellet was resuspended in saline solution and placed onto slides. To determine the differential leukocyte counts represented by the subpopulations of lymphocytes, monocytes, and granulocytes (%), BALF cells were stained with azur-eosin using the Romanovsky–Giemsa method and examined by optical microscopy.

### 2.4. Blood Serum Preparation

Blood samples were collected via retro-orbital sinus puncture in standard test tubes using heparinized microcapillary tubes. Blood serum was prepared from whole blood via clot formation at 37 °C for 30 min, followed by overnight incubation at 4 °C, clot removal, and centrifugation at 4000 rpm at 4 °C for 20 min to eliminate cell debris. Serum samples were stored at −70 °C.

### 2.5. Isolation of Spleen Neutrophils

Spleen neutrophils were isolated via positive selection using anti-LY6G (Purified Anti-Mouse Ly6G Monoclonal Antibody E-AB-F1108A, Elabscience Biotechnology, Houston, TX, USA) and Dynabeads Sheep anti-Rat IgG (Invitrogen, San Diego, CA, USA), as described previously [18]. The quality and viability of neutrophils were assessed by flow cytometry using Annexin V-FITC/PI staining (Apoptosis Detection Kit, Vazyme, Nanjing, China).

### 2.6. Isolation of Total RNA

RNA was isolated from neutrophils using Rizol (diaGene, Moscow, Russia) according to the manufacturer’s guidelines. The quality and concentration of the isolated RNA were evaluated using a NanoDrop™ D-1000 spectrophotometer (ThermoFisher Scientific, Waltham, MA, USA).

### 2.7. cfDNA Isolation

cfDNA was extracted from blood serum and BALF samples using the DNeasy Blood & Tissue Kit (Qiagen, Germantown, MD, USA) according to the manufacturer’s instructions. The concentration of cfDNA was quantified using a Qubit fluorometer (Invitrogen, Carlsbad, CA, USA) with a Quant-iT dsDNA HS Assay Kit (Thermo Fisher Scientific, Waltham, MA, USA), according to the manufacturer’s guidelines. The quality of cfDNA was assessed using a NanoDrop™ ND-1000 spectrophotometer (ThermoFisher Scientific, Waltham, MA, USA).

### 2.8. Assessment of DNase Activity in Blood Serum and BALF

Total DNase activity in blood serum was measured by cleaving the plasmid pHIV. A reaction mixture of 30 μL, consisting of 1 μL of serum or BALF and 0.5 μg of pHIV, was incubated at 30 °C for 5–30 min. The resulting cleavage products were analyzed by electrophoresis on a 1% agarose gel [diaGene, Moscow, Russia] and stained with ethidium bromide [Invitrogen, Carlsbad, San Diego, CA, USA], and the effective cleavage rate constants (keff) were determined as previously described. [19].

### 2.9. qPCR Analysis of Quantitative Content of Nuclear and Mitochondrial DNA in cfDNA

Amplification was performed in 20 μL of reaction mixture containing 0.1–0.5 ng of DNA, SYBR-Green-containing Bio Master CorHS-qPCR (BiolabMix, Novosibirsk, Russia), and 0.6 μM of each of the forward and reverse specific primers for nuclear (*Gapdh*, *Actb*) and mitochondrial (*mt-Nd3*, *mt-Co3*) genes. The primer sequences (Institute of Chemical Biology and Fundamental Medicine SB RAS (Novosibirsk, Russia) are listed in Table 1. PCR was performed using a CFX96 Touch Real-Time PCR Detection System (Bio-Rad Laboratories Inc., Hercules, CA, USA). The reaction conditions were as follows: 95 °C, 6 min; 95 °C, 15 s; 60 °C, 20 s; 70 °C, 60 s; 40 cycles. The sequence of the LINE *element L1_mus1* was used as a reference.

### 2.10. RT-qPCR Analysis of Expression of Genes Characterizing the Pro- and Anti-Inflammatory Phenotype of Neutrophils

cDNA was prepared in 40 μL of reaction mixture containing 2 μg total cellular RNA, 5× RT buffer mix (BiolabMix, Novosibirsk, Russia), 200 U reverse transcriptase MMuLV-RH (Biolabmix, Novosibirsk, Russia), 1 μM (final concentration) primer dT18, and 1 μM (final concentration) random hexamers primer (Institute of Chemical Biology and Fundamental Medicine SB RAS, Novosibirsk, Russia. Reverse transcription was performed as follows: 42 °C for 1 h; 70 °C for 10 min.

The reaction mixture for qPCR (12,5 μL) contained 12.5 ng of cDNA, BioMaster HS-qPCR (BiolabMix, Novosibirsk, Russia), 0.4 μM of each of the forward and reverse specific primers, and 0.25 μM of probes (Table 2). The reaction conditions were as follows: 95 °C, 6 min; then 45 cycles 95 °C, 15 s, 56 °C, 20 s, 70 °C, 60 s. *Hprt1* was used as a reference. The data were analyzed using the *ΔΔCt* method.

### 2.11. Histology and Immunohistochemistry

Lung samples (*n* = 9 for each group) were fixed in 10% buffered formalin (BioVitrum, Moscow, Russia), dehydrated in increasing concentrations of ethanol and xylene, and embedded in HISTOMIX paraffin (BioVitrum, Saint Petersburg, Russia). Paraffin sections up to 5 µm thick were cut using a Microm HM 355S microtome (Thermo Fisher Scientific, Waltham, MA, USA) and stained with hematoxylin and eosin. Connective tissue fibers were assessed using Van Gieson’s staining.

For immunohistochemical study, the lung sections (*n* = 3 for each group) were deparaffinized and rehydrated. Antigen retrieval was carried out after exposure in a microwave oven at 700 W. The samples were incubated with anti-collagen I primary antibodies (AF0134, Affinity Biosciences, Cincinnati, OH, USA) according to the manufacturer’s protocol. Then, the sections were incubated with secondary horseradish peroxidase (HPR)-conjugated antibodies, exposed to the 3,30-diaminobenzidine (DAB) substrate (Rabbit Specific HRP/DAB (ABC) Detection IHC Kit, ab64261, Abcam, Boston, MA, USA) and stained with Mayer’s hematoxylin.

The intensity of inflammatory and fibrotic changes, as well as collagen I expression in the lungs, was assessed semiquantitatively using the following scale: 0—no pathological changes; 1—mild inflammation/fibrosis/collagen I expression; 2—moderate inflammation/fibrosis/collagen I expression; and 3—severe inflammation/fibrosis/collagen I expression. In total, 10 visual fields were analyzed for each sample (in total 90 visual fields in each group for histology and 30 visual fields in each group for immunohistochemistry).

Histological images were examined and scanned using an Axiostar Plus microscope equipped with an AxioCam MRc5 digital camera (Zeiss, Oberkochen, Germany) at magnification ×200.

### 2.12. Statistical Analysis

All experiments were replicated three times. Data were statistically analyzed using a two-tailed unpaired Student’s *t*-test and one-way ANOVA. Post hoc testing was completed using the post hoc Fisher (histology data) or Tukey’s test (in vitro and qPCR data); *p* < 0.05 was considered statistically significant. The statistical package STATISTICA version 10.0 was used for the analysis.

## 3. Results

### 3.1. The Effect of Pulmozyme on the Development of Inflammation and Fibrosis in LPS-Induced Mouse Model

**Figure 1:**
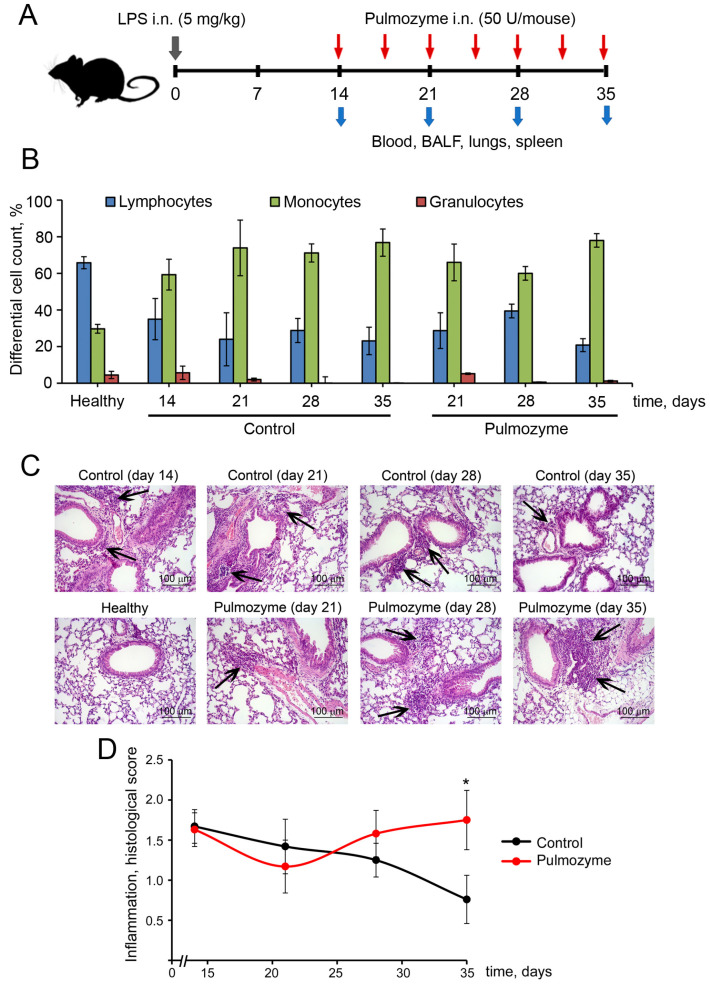
Inflammatory changes in the respiratory system of mice with LPS-induced inflammation/fibrosis without treatment and after Pulmozyme administration. (**A**). Experimental scheme. LPS (5 mg/kg) was singly instilled intranasally (i.n.) in mice and, starting from day 14 after induction, Pulmozyme (50 U/mouse) was administered i.n. twice a week. On days 14, 21, 28, and 35 after LPS challenge, peripheral blood, bronchoalveolar lavage fluid (BALF), lungs, and spleens were collected for subsequent analysis. (**B**). Differential leukocyte counts in BALF of control and experimental mice. (**C**). Representative histological images of lungs of control and experimental mice. Hematoxylin and eosin staining. Original magnification ×200. Black arrows indicate inflammatory infiltration in the lung tissue. (**D**). The intensity of inflammation in the lungs of the control (black curves) and experimental (red curves) groups assessed by a semiquantitative method, where 0—no pathological changes, 1—mild inflammation, 2—moderate inflammation, 3—severe inflammation. Statistically significant differences at * *p* < 0.05 relative to the control.

A mouse model of LPS-induced pulmonary inflammation with successive fibrosis development was used to evaluate the anti-inflammatory and antifibrotic effects of Pulmozyme. The LPS-induced model comprising inflammation-mediated destruction of the lung tissue matrix with subsequent replacement of damaged sites with fibrotic tissue is optimal for studying anti-inflammatory and antifibrotic activity of different compounds, including DNA-targeted drugs. LPS at a dose of 5 mg/kg was singly instilled intranasally (i.n.) in mice and, starting from day 14 after induction, Pulmozyme at a dose of 50 U/mouse was administered i.n. twice a week. On days 14, 21, 28, and 35 after LPS administration, peripheral blood, bronchoalveolar lavage fluid (BALF), lungs, and spleens were collected. The experimental design is shown in Figure 1A.

The BALF cell composition of healthy mice was represented predominately by lymphocytes (65.8%), macrophages (29.7%), and, to a much lesser extent, granulocytes (4.5%) (Figure 1B, Table S1). As expected, on day 14 after LPS administration, the number of macrophages in the BALF of LPS-challenged mice was 2-fold increased, with a slight elevation in the number of granulocytes compared with healthy animals. Subsequently, macrophage counts remained unchanged throughout days 21–35 after induction, but the granulocyte fraction disappeared (Figure 1B, Table S1). In the BALFs of the Pulmozyme-treated group, macrophage counts were insignificantly decreased by days 21–28 compared with the control group, but a certain rise of this cell population, reaching values similar to those of the untreated control, was observed on day 35 after induction, indicating the possible development of secondary inflammation in the respiratory system of mice. An interesting observation was the 2.6-fold elevation of BALF granulocytes on day 21 in the Pulmozyme-treated group compared with the control group, which nevertheless remained at the level of inflammation before treatment initiation (day 14) (Figure 1B, Table S1). Some amount of granulocytes was detected in the BALF of Pulmozyme-treated animals until days 28–35, whereas this cell fraction disappeared entirely from the control group, additionally indicating the persistence of inflammation in the lungs upon Pulmozyme administration. It should be noted that all differences in the cell composition of BALFs were statistically insignificant and only trends were observed.

Histological examination of lung tissue from LPS-challenged mice confirmed persistent inflammation in the Pulmozyme-treated group. As shown in Figure 1C, intranasal administration of LPS led to the development of inflammatory changes in the lungs expressed moderately on day 14 after induction (time point of the treatment start), represented by lymphocytic and macrophage infiltration with a slight neutrophil admixture, as well as edema and vascular congestion. These changes were most obvious on days 14 and 21 and gradually diminished by days 28–35 after induction (Figure 1C). Pulmozyme administered intranasally did not significantly affect the intensity of inflammation until day 28, but on day 35, it caused a 2.7-fold increase in this parameter compared with the control (Figure 1C,D). It should be noted that on day 35, the level of inflammation in the Pulmozyme-treated group was close to that on day 14 (start of treatment).

**Figure 2:**
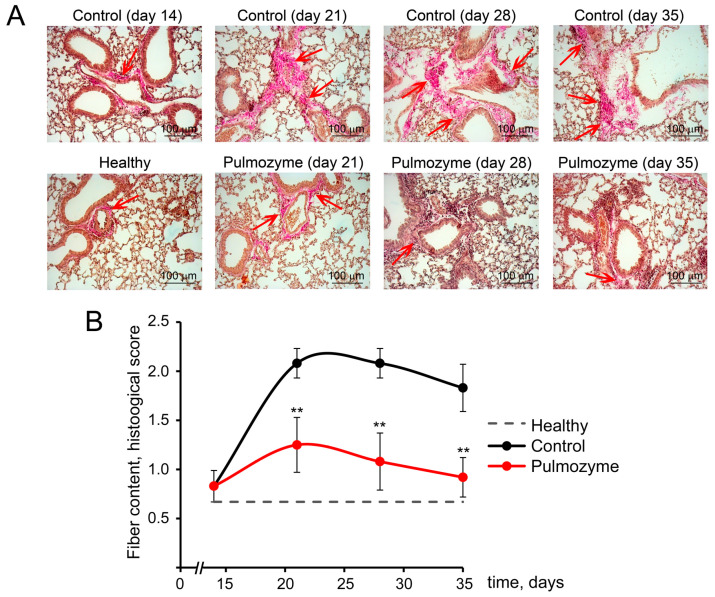
Fiber content in the lung tissue of LPS-challenged mice without treatment and after Pulmozyme administration. (**A**). Representative histological lung images of the control and experimental groups. Van Gieson’s staining. Original magnification ×200. Red arrows indicate connective tissue growth in the lungs. (**B**). The effect of Pulmozyme on the fiber content in the lungs. The intensity of fiber expansion in the lungs of the control (black curves) and experimental (red curves) groups was assessed by a semiquantitative method, where 0—no pathological changes, 1—mild fiber expansion, 2—moderate fiber expansion, and 3—severe fiber expansion. The dotted lines show fiber content in the lungs of healthy animals. Statistically significant differences at ** *p* < 0.001 relative to the control.

When assessing connective tissue growth in the lungs, we detected a small number of stained fibers in healthy mice (Figure 2A,B). A single i.n. administration of LPS caused proliferation of connective tissue fibers up to day 21 after induction, which remained at the same level until day 35, reflected in 3.2-, 3.2-, and 2.7-fold increases in the fiber content in lung tissue on days 21, 28, and 35, respectively, compared with the healthy animals (Figure 2A,B). i.n. instillations of Pulmozyme prevented LPS-induced fiber expansion and led to 1.7-, 1.9-, and 2-fold decreases in the connective tissue growth in the lungs of LPS-challenged mice on days 21, 28, and 35 after induction, respectively, compared with the control (Figure 2A,B).

**Figure 3:**
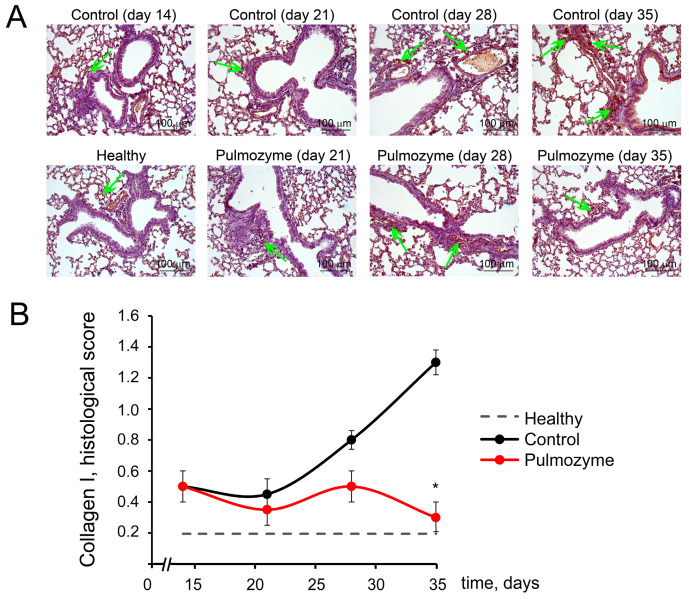
Collagen I expression in the lung tissue of LPS-challenged mice without treatment and after Pulmozyme administration. (**A**). Representative immunohistochemical images of the lung sections of the control and experimental groups stained with anti-collagen I primary antibodies. Original magnification ×200. Green arrows indicate collagen I in the lungs. (**B**). The effect of Pulmozyme on collagen I expression in the lungs. The intensity of collagen I expression in the lungs of the control (black curves) and experimental (red curves) groups was assessed by a semiquantitative method, where 0—no pathological changes, 1—mild collagen I expression, 2—moderate collagen I expression, and 3—severe collagen I expression. Dotted lines show collagen I expression in the lungs of healthy animals. Statistically significant differences at * *p* < 0.05 relative to the control.

The positive effect of Pulmozyme regarding pulmonary fibrosis development was confirmed by collagen I immunohistochemical staining (Figure 3). As can be seen from Figure 3, healthy lungs are characterized by weak expression of collagen I. LPS administration caused an increase in the collagen amounts, especially around large vessels and bronchi, by 4 and 6.5 times on days 28 and 35 after induction compared with the healthy animals (Figure 3A,B). Pulmozyme prevented such a significant increase in collagen expression in the lung tissue, causing 1.5-, 1.6-, and 4.3-fold decreases in this parameter on days 21, 28, and 35 after LPS challenge compared with the control (Figure 3A,B). However, statistically significant differences between the control and treatment groups were detected only on day 35 of the experiment.

Thus, we observed significant attenuation of LPS-induced pulmonary fibrosis by Pulmozyme. However, the expected resolution of inflammation in the respiratory system of the mice did not occur, which could be associated with the functional activity of neutrophils preventing the expansion of connective tissue fibers in lung parenchyma.

### 3.2. The Effect of Pulmozyme on the Concentration and Composition of cfDNA and DNase Activity in Blood Serum and BALF of Mice with LPS-Induced Inflammation/Fibrosis

Considering the fact that granulocytes were mostly present in the BALF, while in the lung tissue only single cells were identified, we assumed that granulocytes temporarily migrate to the lungs, where they either expire or migrate back. One of the common mechanisms of neutrophil cell death is NETosis, followed by the release of NETs, which significantly enrich the fraction of extracellular DNA. Pulmozyme, being a DNase I preparation, should reduce the concentration of extracellular DNA and, at the same time, increase DNase activity in the blood or BALF of treated mice.

To evaluate the effect of Pulmozyme based on its ability to destroy DNA, cell-free DNA (cfDNA) concentrations and DNase activity of blood serum and BALF were measured. The data obtained are summarized in Table 3.

In the blood serum of control group animals, the levels of cfDNA, as well as DNase activity, were low throughout the experiment and did not differ from those of healthy animals (Table 3). Pulmozyme administration caused a 3-fold increase in the level of cfDNA in blood serum on days 21 and 28 and more than a 17-fold increase on day 35, while DNase activity of blood serum increased slightly (1.2-fold) up to day 35 compared with the control (Table 3).

Surprisingly, the concentration of cfDNA in the BALF was 1000-fold higher than that in blood serum, even in healthy mice. After LPS-induced inflammation, the level of cfDNA in BALF increased 2.8-fold relative to healthy animals on day 14 and 3.5-fold on days 21 and 28; then, some decrease in cfDNA levels on day 35 was observed (Table 3). A similar tendency for cfDNA levels in BALF was observed in Pulmozyme-treated mice. As for DNase activity, it was 1.5 times lower than in blood serum in the BALF of the control group (Table 3). Pulmozyme administration resulted in an increase in DNase activity in the BALF relative to the control on day 21, and this activity remained at a similar level until the end of the experiment.

The principal limitation of DNase I, the main component of Pulmozyme, is the ability to cleave only naked DNA, free of proteins and lipid membranes. Taking into account the fact that mitochondrial DNA is released from dying cells in the form of membrane particles, we analyzed the ratio of mitochondrial (*mt-Nd3*, *mt-Co3*) and nuclear (*Actb*, *Gapdh*) genes in cfDNA samples from BALF using qPCR. The L1_mus1 sequence, located in heterochromatin and inaccessible for degradation by DNase, was used as reference.

**Figure 4:**
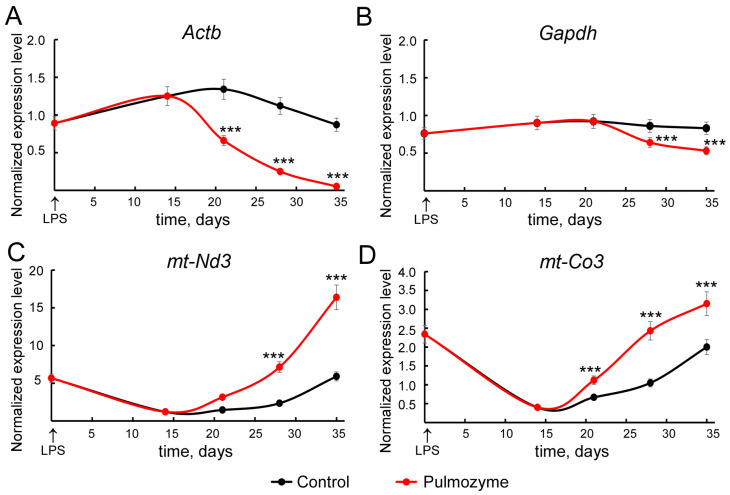
Amounts of nuclear (*Actb* and *Gapdh*, (**A**,**B**)) and mitochondrial (*mt-Nd3* and *mt-Co3*, (**C**,**D**)) sequences in BALF cfDNA from LPS-challenged mice without treatment (control, black curves) or after Pulmozyme administration (red curves). The number of copies of specific gene sequences was normalized to the number of L1_mus1 sequences. Data from RT-qPCR analysis. Statistically significant differences at *** *p* < 0.0001 relative to the control.

Interestingly, the levels of nuclear and mitochondrial gene sequences exhibited different tendencies upon the development of LPS-induced inflammation, both without treatment and after Pulmozyme administration. It was shown that in control BALF samples, a higher accumulation of nuclear gene sequences relative to mitochondrial ones was observed on days 14–21 after induction (Figure 4A–D, black curves). However, from day 28 and until the end of the experiment, there was an increase in the content of mitochondrial gene sequences and some reduction in nuclear gene sequences in the BALF of control mice. Interestingly, at the end of the experiment, the levels of both nuclear and mitochondrial sequences were close to those in the healthy control group (Figure 4A–D, black curves). In BALF samples from animals treated with Pulmozyme, a pronounced accumulation of mitochondrial sequences and reduction in the number of nuclear sequences throughout the experiment were observed (Figure 4A–D, red curves).

Obtained results confirm that Pulmozyme efficiently cleaves the nuclear DNA fragments present in BALF of LPS-challenged mice, leaving mitochondrial ones unaffected.

### 3.3. Effect of Pulmozyme on the Neutrophil Profile in the Spleen of Mice with LPS-Induced Inflammation/Fibrosis

The spleen is a depot for various immune cells and is also a site of secondary migration for certain types of cells, including neutrophils [20,21]. Considering that neutrophils are active participants in fibrotic processes, it seemed relevant to evaluate the dynamics in the neutrophil profile after treatment with Pulmozyme. For this purpose, total RNA from neutrophils obtained from the spleens of healthy mice, control mice, and mice treated with Pulmozyme was isolated. The expression of various genes was assessed using RT-qPCR.

**Figure 5:**
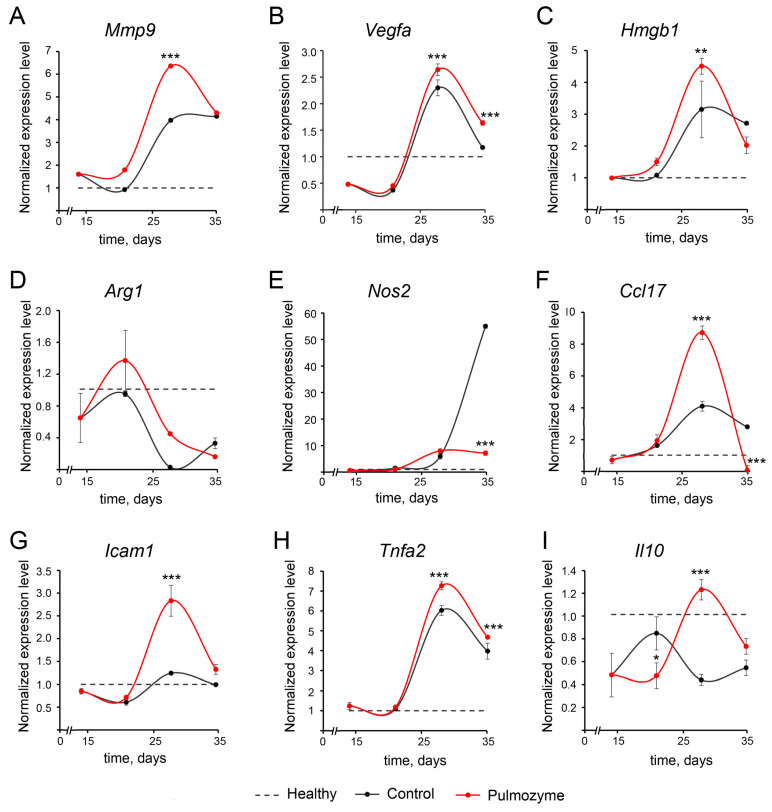
The gene profiles in the spleen-derived neutrophils of healthy and LPS-challenged mice without treatment (control, black curves) and after Pulmozyme administration (red curves). (**A**). *Mmp9*. (**B**). *Vegfa*. (**C**). *Hmgb1*. (**D**). *Arg1*. (**E**). *Nos2*. (**F**). *Ccl17*. (**G**). *Icam1*. (**H**). *Tnfa.* (**I**). *Il10*. RT-qPCR data. *Hprt* was used as the reference gene. Dotted lines show the level of respective gene expression in healthy animals. Data were analyzed using the nonparametric statistical test ANOVA with the Tukey’s post hoc test. Statistically significant differences at * *p* < 0.05, ** *p* < 0.001 and *** *p* < 0.0001 relative to the control.

To analyze the neutrophil properties, we used the following frequently mentioned profiles: pro-tumor anti-inflammatory profile (*Mmp9*, *Vegfa*, *Arg1*, *Nos2*, *Ccl17*, *Il10*) and anti-tumor pro-inflammatory profile (*Icam1*, *Tnfa*) [22,23,24]. In addition, we examined the mRNA levels of *Hmgb1*, which indicates the general mobilization of cellular processes and participates in TLR9 signaling in response to DNA stimulation [25], and *Col1a1*, the expression of which indicates the transition of neutrophils into fibroblast-like cells [26], occurring in various fibrotic processes [27]. Obtained data are displayed in Figure 5.

As seen above, up to day 21 in both the control and experimental (Pulmozyme-treated) groups, the expression levels of investigated genes were similar (*Mmp9*, *Hmgb1*, *Nos2*, *Ccl17*, *Tnfa*) or somewhat lower (*Vegfa*, *Arg1*, *Icam1*, *Il10*) than those in healthy species (Figure 5). The main changes in gene expression were observed during days 28–35, with the exception of *Arg1* and *Nos2*, which exhibited entirely different expression profiles.

On day 28 in both the control and experimental groups, an accumulation of neutrophils with increased expression levels of genes of potential pro-fibrotic phenotype, namely *Mmp9*, *Vegfa*, *Ccl17*, and *Tnfa*, as well as important nuclear regulator *Hmgb1*, was observed (Figure 5A–C,F,H). It is worth mentioning that in the experimental group, on day 28, the expression levels of *Ccl17* (2-fold), *Mmp9* (1.5-fold), *Hmgb1* (1.5-fold), and *Tnfa* (1.2-fold) were higher than in the control group, while the level of *Vegfa* was comparable in both groups.

*Icam1*, *Il10*, *Arg1*, and *Nos2* expression profiles were very distinct compared to the rest. In the control group, the level of *Icam1* remained unaffected, and the level of *Il10* was almost 2-fold lower than in healthy mice. After Pulmozyme administration, a 3-fold increase in *Icam1* level was observed up to day 28, with a sharp drop to the level of healthy mice on day 35, while the level of *Il10* increased up to the level of healthy mice on day 28 and then again dropped down to the control group level on day 35 (Figure 5G,I). *Arg1* and *Nos2* expression levels demonstrated opposite dynamics. The *Arg1* expression level sharply decreased compared to the healthy mice by day 28 and remained low until the end of the experiment, while *Nos2* expression level was significantly (up to 10-fold) increased from day 28 to day 35, but only in the control group. The *Nos2* expression level after Pulmozyme treatment did not differ significantly from the healthy group (Figure 5D,E).

The main differences between neutrophils from the control and experimental groups were as follows: significantly elevated expression levels of *Ccl17*, *Hmgb1*, *Mmp9*, *Icam1*, and *Il10* on day 28; and very low intensity of *Nos2* activation. These results are evidence that the neutrophil population in the experimental group is enriched by the neutrophils with mixed properties (or, more likely, consists of different populations), which cannot be fully attributed to either the N1 or N2 phenotype due to several reasons. First is that the elevated expression of *Il10* indicates potential broad-spectrum anti-inflammatory functions [28,29]; second, the expression of *Icam1*, one of the adhesion molecules, suggests increased extravasation activity [30]; and third, the high expression levels of *Mmp9* and *Hmgb1* indicate the increased potential for remodeling of the surrounding space [31,32,33] and potency for mobilization of cellular processes (including the ability to perform DAMP functions), respectively [34]. The expression level of *Vegfa*, which is associated with increased readiness for vascular remodeling, was significantly higher compared to healthy mice and was observed in both the control and experimental groups [35]. Surprisingly, increased expression of *Ccl17*, a pro-inflammatory cytokine whose main functions are attraction of type 2 T-helpers and, to a lesser extent, leukocytes [36], observed in the experimental group, may partly explain the secondary inflammation that occurs after Pulmozyme treatment.

## 4. Discussion

Pulmonary fibrosis is a chronic progressive lung disorder that inevitably leads to lung architecture disruption and respiratory failure [1]. The development of pulmonary fibrosis can be the result of previous acute or chronic lung inflammation, involving a wide variety of inflammatory cells and causing increased deposition of connective tissue fibers in the lungs. It was shown [37,38] that the most pronounced inflammatory changes in the respiratory system of mice after i.n. LPS administration (acute lung injury) were observed at 24 h after induction, decreasing at 72 h. As can be seen from Figure 1 and as shown previously [39], acute inflammation subsides by day 14 after induction, remaining at a moderate level for a long time. However, exactly at this time interval (14 days), the development of initial fibrotic changes in the lungs can be detected. For this reason, we started antifibrotic therapy at that time point (day 14 after induction), considering the fact that, at this time point, the further course of inflammation is decided: either resolution and healing of the affected lung tissue or transition to the chronic form and fibrotic transformation.

It has been repeatedly shown that neutrophil recruitment followed by neutrophil extracellular trap (NET) release with subsequent neutrophil death, so-called NETosis, is an important factor for fibroblast-to-myofibroblast transformation, a key step of fibrosis development [40]. A growing body of evidence indicates that NETs and their individual components contribute significantly to the progression of pulmonary fibrosis, including the DNA core strands themselves [12,41,42], histones [43], components of neutrophil granules (e.g., MPO, NE) [6,41,44], HMGB1 [45], and others.

It has been suggested that the use of cytokine inhibitors that reduce neutrophil recruitment, as well as NETosis inhibitors (e.g., PAD4 inhibitors), may be promising antifibrotic drugs [40]. In particular, the PAD4 inhibitor effectively reduced the fibrotic and inflammatory effects of bleomycin-induced lung injury [46]. In vitro experiments have shown that bovine pancreatic DNase I effectively destroys NETs and ameliorates their transforming effects on fibroblasts [12]. Therefore, we hypothesized that human recombinant DNase I in the stable form of Pulmozyme, adapted for topical administration and poorly absorbed into the blood when administered by inhalation in humans [47], would be an effective agent against neutrophil-dependent inflammation and fibrosis.

In our study, i.n. instillations of Pulmozyme were performed on day 14 (fibrosis start) and regularly repeated up to day 35 after LPS challenge. It was found that topical administration of Pulmozyme in this phase of LPS-induced inflammation, when acute changes have already subsided and chronic ones have just begun to form, effectively prevents the expansion of connective tissue in the lungs, providing a significant antifibrotic effect. However, some signs of secondary inflammation after Pulmozyme administration (days 28–35 after LPS-challenge) were observed. In should be noted that the intensity of the inflammation was higher in the Pulmozyme-treated group despite its reliable antifibrotic effect, but was also observed in the control group to a smaller extent (Figure 1C,D).

We found that the most unexpected events accompanying Pulmozyme treatment were the persistent nature of inflammation in the lung tissue, the elevated level of mitochondrial DNA sequences in the BALF, and the unclassified gene expression profile of splenic neutrophils, presumably close to the classical N2 pro-tumor phenotype observed from day 21 and reaching its maximum by day 28 after induction. The positive effect of Pulmozyme was to reduce fibrotic changes in the lungs of mice, namely the growth of connective tissue and collagen density.

We suggest that the main mechanism underlying the antifibrotic action of Pulmozyme is the disruption of DNA in the extracellular matrix (ECM) of the lungs and BALF. Analysis of cfDNA in the BALF of mice after LPS induction showed its high concentration, 1000 times higher than that in the blood serum. These data correlated well with NET enrichment of sputum and lungs of patients with ARDS [10], COPD [11], and non-specific interstitial pneumonia [12]. The suggested mechanism is supported by our data showing a slight decrease in the concentration of cfDNA and an increase in DNase activity in the BALF of LPS-challenged mice after treatment with Pulmozyme. At the same time, we did not observe any effect of Pulmozyme on DNase activity or the cfDNA amount in the blood, which confirms poor penetration of the enzyme (DNase I, the main component of Pulmozyme) into the blood after topical administration [47].

As for the observed signs of secondary inflammation in the respiratory system upon Pulmozyme treatment, we assume that such effects could be attributed to the changes in the composition of the cfDNA. We assessed the ratio of nuclear and mitochondrial DNA in BALF and found that Pulmozyme significantly decreased the levels of nuclear but not mitochondrial DNA sequences. Some studies have shown that the presence of mitochondrial DNA is an important factor promoting inflammation [48,49,50]. According to the literature, mitochondrial DNA can be secreted in the form of vesicular particles, which complicates the extracellular degradation of DNA but promotes the activation of some cell types [51]. Some stimuli, including LPS and fibrin, promote the release of NETs containing mitochondrial DNA, conferring greater stability and additional resistance to nucleases [52,53]. According to our results, DNase I in Pulmozyme cannot effectively degrade mitochondrial DNA, which apparently, with the disappearance of the nuclear component of cfDNA, becomes an additional factor contributing to secondary inflammation. However, this assumption requires further investigations.

An interesting observation was the recruitment of lung granulocytes into the BALF upon Pulmozyme treatment. In the control group, this cell fraction had already disappeared by day 21 after induction. In the experimental group, granulocytes were detected in BALF up to day 35. Considering that neutrophils and other granulocytes are multifunctional cells, they perform different functions at different stages of fibrotic processes and therefore have different phenotypes [54]. Since the study of the profile of even neutrophils, the major fraction of granulocytes, is difficult due to the small number of cells for the differential staining of markers, it was decided to analyze the profile of neutrophils in the spleen as the main depot of immature and naive neutrophils, and the secondary migration site for mature and functionally active ones [18].

The results of this study showed that in the control and experimental groups, neutrophils that accumulated in the spleen in the later stages of fibrosis (28–35 days) possessed pro-fibrotic properties and demonstrated a high expression level of matrix metalloproteinase *Mmp9*, which is involved in ECM remodeling, the increased mRNA level of *Vegfa*, which promotes vascular growth, and the increased mRNA level of pro-inflammatory factor *Tnfa*. As observed in the control group, the level of *Hmgb1* mRNA was increased, which could explain the enhanced secretion of Mmp9, Vegfa, and Tnfa [55]. Thus, co-expression of *Hmgb1*, *Mmp9*, and *Vegfa* may reflect the increased regenerative activity of neutrophils and their involvement in tissue remodeling [56]. Interestingly, after treatment with Pulmozyme, neutrophils with the same profile were found in the spleen; however, they demonstrated greater regenerative abilities with significantly higher expression levels of *Mmp9* and *Hmgb1* than in the control group. The levels of *Vegfa* and *Tnfa* remained unaffected in both groups but were significantly higher than those in the healthy control.

The above-mentioned factors, as well as the increased expression level of pro-inflammatory *Ccl17* involved in neutrophil recruitment, may explain the persistent inflammation observed in the lung tissue of Pulmozyme-treated mice. Since CCL17 can exhibit opposite effects on cells involved in lung fibrosis development, activating the TGF-β/Smad signaling pathway, and promoting fibroblast activation [23], the overall significance of *Ccl17* overexpression remains unclear, and the presence of neutrophils in the spleen with increased expression of this cytokine requires further investigation.

One of the observations was an increase in *Il10* expression levels after treatment with Pulmozyme. *Il10* is widely known for its anti-inflammatory properties [57], but also has antifibrotic effects, as evidenced by its ability to suppress collagen I production in human fibroblasts [58]. The therapeutic relevance of IL10 has been demonstrated [59,60], and its hydrogel-based delivery improves bleomycin-induced lung fibrosis in mice [61]. LPS challenge decreased the expression of *Il10* throughout the experiment, but Pulmozyme administration restored the mRNA level of this cytokine, which may indicate a switch in the neutrophil profile from pro-inflammatory (control group) to anti-inflammatory (experimental group).

Interestingly, neutrophils in the spleens of the Pulmozyme-treated group actively expressed *Icam1* mRNA, which may indicate increased neutrophil migratory properties. An enhancement of *Icam1* expression most often occurs upon acute inflammation and viral infections involving functionally active neutrophils with an increased ability to migrate, which can be additional factor of observed persisting inflammation [62]. An increase in ICAM1 levels may often indicate activation of the migratory properties of neutrophils [63]. However, in the case of inflammatory reactions, the situation is somewhat more complicated: ICAM1 and ICAM2 promote homotypic aggregation and adhesion [30,64] but are apparently not required for cell migration to the site of inflammation [30,65]. Nevertheless, high ICAM 1 is required for phagocytosis of murine neutrophils and also promotes ROS stimulation in inflammatory reactions [30,66,67].

The relationship between *Arg1* and *Nos2* expression levels deserves attention. Both of these factors are supposed to be present in anti-inflammatory and pro-tumor neutrophils [24]. However, the interplay between the products of these two genes appears to be more complex. Arginase levels decreased dramatically in the control group on day 28 after induction. This may indicate a high level of arginine, which induces the expression of *Nos2* observed at a later stage of inflammation (day 35, control group). In the literature, arginase inhibition enhances the activity of NOS1, one of the NOS isoforms [68]. High arginase activity results in low plasma L-arginine and nitric oxide levels, leading to increased inflammation and airway remodeling [69]. In the Pulmozyme-treated group, low *Arg1* levels accompanied by low *Nos2* were detected, which can be a predictor for further resolution of residual inflammation in the lung tissue of experimental animals. Overall, it can be speculated that spleen-derived neutrophils in the Pulmozyme-treated group have a very distinct profile, which cannot be called either pro-inflammatory or anti-inflammatory. However, there is no doubt that such neutrophils should have an increased capacity for the attenuation of ECM remodeling, partially explaining the antifibrotic effect of Pulmozyme.

Thus, it was clearly shown that Pulmozyme significantly ameliorates LPS-induced lung fibrosis but maintains residual inflammation in the respiratory system of mice, possibly due to modulation of the cfDNA composition in BALF and enrichment of the neutrophil population in the spleen by those with an unusual phenotype, combining pro-inflammatory and anti-inflammatory features. The distant events in the lungs after LPS challenge, modeling human inflammation-associated lung diseases, require further investigation. However, our findings regarding the cfDNA profile in BALF, neutrophil populations in the spleen, and the fact that inflammation in the lungs is maintained but not exacerbated after Pulmozyme administration let us suggest that after treatment the lungs will recover without residual fibrotic changes.

## 5. Conclusions

In this study, we have shown that treatment with Pulmozyme resulted in a noticeable reduction in fibrotic transformation in a mouse model of LPS-induced lung fibrosis. Positive effects of Pulmozyme were accompanied by signs of residual inflammation with additional granulocyte recruitment in the BALF, as well as elevation of mitochondrial DNA sequences of cfDNA. In the spleen, accumulation of neutrophils with a mixed phenotype occurred, reflecting potential abilities for its regenerative capacity (increased mRNA levels of *Mmp9*, *Hmgb1*, *Ccl17*, *Icam1*, *Il10*, and decreased *Nos2*). In total, the observed secondary inflammation may be both an independent phenomenon and one of the factors contributing to the antifibrotic action of Pulmozyme.

### Limitations

In this study, serum, not plasma, samples were used to isolate cfDNA. Neutrophil samples for RNA extraction were pooled for data averaging. Additionally, neutrophil phenotype was analyzed by RT-qPCR reaction, and while it allows detection of the exact levels of gene expression at the mRNA levels, it cannot be used for the analysis of protein levels and individual cell populations.

## Figures and Tables

**Table 1:** Primer sequences for qPCR (DNA sequence detection).

Gene	Primer Sequence, 5′→3′	
	Forward	Reverse
*Actb*	GAGGTATCCTGACCCTGAAGTA	TCTACAATGAGCTGCGTGTG
*Gapdh*	CGCCCTGATCTGAGGTTAAAT	TACACACATCTGTTGCTCCG
*mt-Co3*	CAGGATTCTTCTGAGCGTTCTAT	ACTTAACCCTCTAGAAGTCCCA
*mt-Nd3*	AATGCGGATTCGACCCTAC	CTTCTACTTCCACTACCATGAGC
*L1_mus1*	GCCAGGTATCTGTGCATCTT	ACTCTAGCTCTCTCCTGAGTTT

**Table 2:** Primer and probe sequences for RT-qPCR (RNA sequence detection).

Gene	Sequences, 5′→3′		
	Forward	Reverse	Probe
*Arg1*	AAGAATGGAAGAGTCAGTGTGG	GGGAGTGTTGATGTCAGTGTG	FAM-TCTGGCAGTTGGAAGCATCTCTGG-BHQ1
*Ccl17*	CAGACCCCAAAGACAAACATG	GTCACAGGCCGTTTTATGTTG	FAM-TGACCTTCCCGCTGAGGCATT-BHQ1
*Nos2*	TGGAGCGAGTTGTGGATTG	CGTAATGTCCAGGAAGTAGGTG	FAM-CAGCCTCTTGTCTTTGACCCAGTAGC-BHQ1
*Il10*	AACATACTGCTAACCGACTCC	CAAATGCTCCTTGATTTCTGGG	FAM-ATCATTTCCGATAAGGCTTGGCAACC-BHQ1
*Tnfa*	TGGAGTCATTGCTCTGTGAAG	CCTGAGCCATAATCCCCTTTC	FAM-TCTGACCCCTTTACTCTGACCCCTT-BHQ1
*Icam*	GCAGAGGACCTTAACAGTCTAC	TACTTGGCTCCCTTCCGAGACCT	FAM-TACTTGGCTCCCTTCCGAGACCT-BHQ1
*Mmp9*	GACATAGACGGCATCCAGTATC	GTGGGAGGTATAGTGGGACA	FAM-TCGGCTGTGGTTCAGTTGTGGT-BHQ1
*Vegfa*	CCGAAACCATGAACTTTCTGC	CTTCATGGGACTTCTGCTCTC	FAM-CACTGGACCCTGGCTTTACTGCT-BHQ1
*Hmgb1*	GTACCGCCCCAAAATCAAAG	CCTTCTCATACTTCTCCTTCAGC	FAM-TCACCAATGGATAAGCCAGGATGCTC-BHQ1
*Col1a1*	AGCCGCAAAGAGTCTACATG	CTTAGGCCATTGTGTATGCAG	FAM-CCGGAGGTCCACAAAGCTGAACAT-BHQ1
*Hprt1*	CCCCAAAATGGTTAAGGTTGC	AACAAAGTCTGGCCTGTATCC	ROX-CTTGCTGGTGAAAAGGACCTCTCGAA-BHQ2

**Table 3:** Concentration of cfDNA and DNase activity of blood serum and BALF of LPS-challenged mice without treatment (control) and after Pulmozyme administration.

Days	Concentration of cfDNA, ng/mL		DNase Activity, k_eff_, s^−1^	
	Control	Pulmozyme	Control	Pulmozyme
Blood serum				
0 (healthy)	50.0 ± 0.5		5.97 ± 0.11	
14	50.0 ± 0.5	50.0 ± 0.5	5.97 ± 0.08	5.97 ± 0.08
21	50.0 ± 0.5	140.5 ± 56.0	5.97 ± 0.05	6.05 ± 0.07
28	85.2 ± 25.0	145.4 ± 42.8	6.03 ± 0.07	6.08 ± 0.08
35	50.0 ± 0.5	887.1 ± 254.1 ***	6.03 ± 0.11	6.93 ± 0.34
BALF				
0 (healthy)	(17.2 ± 5.3) × 10^3^		3.83 ± 0.13	
14	(48.4 ± 1.7) × 10^3^	(48.4 ± 1.7) × 10^3^	3.87 ± 0.03	3.87 ± 0.03
21	(60.7 ± 1.5) × 10^3^	(44.3 ± 2.2) × 10^3^ *	3.87 ± 0.03	5.43 ± 0.18 **
28	(59.1 ± 2.) × 10^3^	(51.1 ± 2.8) × 10^3^	3.90 ± 0.18	5.53 ± 0.08 **
35	(28.5 ± 3.4) × 10^3^	(27.8 ± 4.9) × 10^3^	3.83 ± 0.06	5.9 ± 0.05 **

DNase activity was measured in the reaction of pHIV plasmid DNA cleavage. Statistically significant differences at * *p* < 0.05, ** *p* < 0.001, and *** *p* < 0.0001 relative to the control.

## Data Availability

The original contributions presented in this study are included in the article. Further inquiries can be directed to the corresponding author.

## Supplementary Materials

The following supporting information can be downloaded at: https://www.mdpi.com/article/10.3390/biom15020298/s1, Table S1: Total leukocyte, lymphocytes, granulocytes and monocytes counts in BALF of control and experimental mice.
