# Supplementary material for: Pulmozyme Ameliorates LPS-Induced Lung Fibrosis but Provokes Residual Inflammation by Modulating Cell-Free DNA Composition and Controlling Neutrophil Phenotype
Source: Biomolecules. 2025 Feb 17;15(2):298. doi: 10.3390/biom15020298 (PMC11853346; doi:10.3390/biom15020298)
Supplement: Supplementary file 1 [file biomolecules-15-00298-s001.zip › biomolecules-3364675-supplementary.pdf]

Pulmozyme ameliorates LPS-induced lung fibrosis but provokes residual inflammation by modulating cell-free DNA composition and controlling neutrophil phenotype

Ludmila A. Alekseeva[1,†], Aleksandra V. Sen'kova[1,†], Khetam Sounbuli[1,2], Innokenty A. Savin[1], Marina A. Zenkova[1] and Nadezhda L. Mironova[1,*]

1 Institute of Chemical Biology and Fundamental Medicine, Siberian Branch of the Russian Academy of Sciences (SB RAS), Lavrentiev Ave., 8, Novosibirsk 630090, Russia; alekseeva.mila.23@yandex.com (L.A.A.); senkova_av@niboch.nsc.ru (A.V.S.); khetam.sounbuli.edu@gmail.com (K.S.); savin_ia@niboch.nsc.ru (I.A.S.), marzen@niboch.nsc.ru (M.A.Z.), mironova@niboch.nsc.ru (N.L.M.)

2 Faculty of Natural Sciences, Novosibirsk State University, Pirogova st., 1, Novosibirsk 630090, Russia; khetam.sounbuli.edu@gmail.com (K.S.)

* Correspondence: mironova@niboch.nsc.ru (N.L.M.)

† These authors contributed equally to this work

Table S1. Total leukocyte, lymphocytes, granulocytes and monocytes counts in BALF of control and experimental mice.

	Days of experiment	Cell count, ×10⁴/mL			
		**Total leucocytes**	**Lymphocytes**	**Granulocytes**	**Monocytes**
**Healthy**		4.2±0.8	2.8±0.5	0.2±0.1	1.2±0.2
**Control**	14	12.2±3.3	4.3±1.5	0.7±0.2	7.2±1.8
	21	9.3±3.1	2.2±0.8	0.2±0.1	6.9±2.2
	28	7.0±1.7	2.0±0.3	0.007±0.001	5.0±1.4
	35	6.1±1.1	1.4±0.3	0.006±0.001	4.7±0.9
**Pulmozyme**	21	7.0±1.7	2.0±0.5	0.4±0.1	4.6±1.1
	28	10.3±2.2	4.1±1.0	0.06±0.01	6.2±1.2
	35	7.1±1.6	1.5±0.3	0.1±0.01	5.5±1.3
